# Effects of music training on executive functions in preschool children aged 3–6 years: systematic review and meta-analysis

**DOI:** 10.3389/fpsyg.2024.1522962

**Published:** 2025-01-15

**Authors:** Yanan Lu, Lin Shi, Ahmad Faudzi Musib

**Affiliations:** ^1^Department of Music, Faculty of Human Ecology, Universiti Putra Malaysia, Serdang, Selangor, Malaysia; ^2^School of Physical Education, Chengdu Sport University, Chengdu, Sichuan, China; ^3^Center for Post-doctoral Studies of Sport Science, Chengdu Sport University, Chengdu, Sichuan, China

**Keywords:** executive functions, preschool children, inhibitory control, working memory, cognitive flexibility

## Abstract

**Background:**

Executive functions is a crucial ability in the early development of preschool children. While numerous studies have found that music training has a favorable effect on children’s executive functions, there is a lack of a consistent perspective on this topic, particularly with regard to the dose–response relationship.

**Methods:**

Systematic searches were conducted of Web of Science, PubMed, Scopus, and China National Knowledge Infrastructure. A random-effects meta-analysis was used to compute standardized mean differences (SMD) and 95% confidence intervals (CI).

**Results:**

In all, 10 studies were included in the meta-analysis, in which children’s music training groups showed significantly improved inhibitory control (SMD = 0.38, 95% CI: 0.16–0.6), working memory (SMD = 0.35, 95% CI: 0.16–0.54), and cognitive flexibility (SMD = 0.23, 95% CI: 0.04–0.42) in comparison with control groups. Subgroup analyses indicated significant improvements relative to the control groups for inhibitory control following music training having a duration of ≥12 weeks (SMD = 0.51, 95% CI: 0.22–0.8), occurring ≥3 times per week (SMD = 0.48, 95% CI: 0.2–0.75), and lasting 20–30 min per session (SMD = 0.42, 95% CI: 0.2–0.63). Significant improvements were seen for working memory following music training having a duration of ≥12 weeks (SMD = 0.42, 95% CI: 0.18–0.65), occurring <3 times per week (SMD = 0.49, 95% CI: 0.06–0.93), occurring ≥3 times per week (SMD = 0.28, 95% CI:0.1–0.47), and lasting 20–30 min per session (SMD = 0.39, 95% CI: 0.16–0.54). Music training significantly improved cognitive flexibility following training having a duration of ≥12 weeks (SMD = 0.22, 95% CI: 0.04–0.41), occurring ≥3 times per week (SMD = 0.19, 95% CI: 0.0–0.39), and lasting >40 min per session (SMD = 0.74, 95% CI: 0.22–1.26).

**Conclusion:**

Music training has a positive effect on inhibitory control, working memory, and cognitive flexibility in preschool children aged 3–6 years. This effect is influenced by certain training factors, including the duration of the intervention period, frequency per week, and length of each session.

**Systematic review registration:**

https://www.crd.york.ac.uk/prospero/#aboutregpage, CRD42024513482.

## Introduction

Executive functions (EFs), also called executive control or cognitive control, refers to the series of top–down cognitive processes necessary for focusing attention. Simply acting automatically or relying on intuition and instincts would make it impossible to accomplish many tasks (Diamond, 2013; Burgess and Simons, 2005; Espy, 2004; Miller and Cohen, 2001). EFs typically includes three components: inhibitory control, working memory, and cognitive flexibility (Diamond, 2013; Hughes et al., 2009; Espy et al., 2011; Logue and Gould, 2014). Inhibitory control refers to the individual’s capacity to actively suppress, regulate, and filter their own thoughts to maintain cognitive integrity. This involves preventing the inclusion or retention of irrelevant information in the short-term memory (Rothbart et al., 2006; Zelazo and Müller, 2002). The regulation of attention, emotion, and behavior through the inhibition of internal or external distractions is intended to effectively accomplish established goals and tasks (Diamond, 2013). The brain temporarily stores and simultaneously accesses a limited volume of information in working memory, enabling the ability to retain and manipulate information over relatively brief periods (D'Esposito and Postle, 2015). Cognitive flexibility refers to the individual’s capacity to switch, rationalize, and adapt to different rules in varying conditions (Diamond et al., 2016). It particularly involves the ability to change perspectives, engage in critical thinking, address problems using multiple perspectives, and effectively navigate unexpected situations.

The development of EFs is closely linked to the maturation of the brain. In early life, the prefrontal lobes and cortex undergo rapid growth and gradual maturation (Diamond, 2002; Zuk et al., 2014). The prefrontal region oversees the individual’s manipulation of information in attempting to accomplish tasks, ensuring that the task is carried out in a well-coordinated, organized, and nonpanicked manner to reach a specific goal. For example, Wager and Smith (2003) found that engagement in different EFs tasks, such as acquiring information and inhibiting irrelevant information, are correlated with activity in distinct brain regions. This helps individuals integrate information before and after tasks and regulate and control their emotions and actions. In addition, neuroimaging research has indicated that various elements of EFs depend on specific regions of the prefrontal cortex (PFC). In particular, the maintenance of information in working memory predominantly engages the lateral PFC (Narayanan et al., 2005). By contrast, the ability to switch between tasks (cognitive flexibility) relies on the medial PFC (Crone et al., 2006), and the inhibitory control responses are dependent on the inferior frontal cortex (IFC) (Aron et al., 2004). Thus, distinct regions in the PFC support different components of goal-oriented behavior.

EFs has a crucial role to play in children’s early development and is strongly associated with their overall cognitive growth. Providing structured assistance in the cultivation of EFs skills during the preschool period can greatly affect subsequent learning outcomes (Diamond, 2012; Blair and Raver, 2014). Numerous studies have demonstrated significant associations between EFs and various aspects of children’s lives, including school readiness and academic achievement (McClelland and Cameron, 2011), physical health (Miller et al., 2011), and occupational success (Bailey, 2007). EFs develops over a wide span of time, with critical milestones being typically observed between the ages of 2 and 5 years (Garon et al., 2008; Best et al., 2009). Best and Miller (Best and Miller, 2010) demonstrated that 4-year-old preschool children could successfully complete both simple and complex inhibitory control tasks, exhibiting significant improvement in task accuracy from ages 5 to 8. Likewise, cognitive flexibility emerges in preschool children at around 2 years old, progressing gradually between the ages of 3 and 5 years (Garon et al., 2008). It is crucial to cultivate EFs in preschool children, as it can positively influence their cognitive abilities and enhance their future academic performance.

Research has suggested that the implementation of a structured educational curriculum promotes the development of EFs in preschool children, leading to enhanced learning and overall development (Diamond and Lee, 2011). Programs to facilitate this include computerized training (Bergman et al., 2011; Thorell et al., 2009), aerobic exercise (Chaddock et al., 2011), school programs (Lillard and Else-Quest, 2006; Riggs et al., 2006), yoga (Razza et al., 2015), and martial arts (Lakes and Hoyt, 2004). Taking into account the consistent and repetitive nature of EFs training (Diamond and Lee, 2011) and considering that music training is characterized by its regularity, enjoyment, and repetition (Miendlarzewska and Trost, 2014), the correlation is clear. During music training, individuals need to pay attention to information arriving through sensory channels, switching between sensory stimuli in real time, integrating information from multiple sensory channels, and storing it in working memory at any time. In addition, individuals must inhibit the interference from other external stimuli that may compete for attention (Moradzadeh et al., 2015; Sato et al., 2015; Slevc et al., 2016).

Several research studies have indicated that music education produces a positive impact on enhancements to the EFs and the cognitive abilities of children. For instance, in their systematic review Degé and Frischen (2022) highlighted the positive effects of music training on 3–14 year olds’ inhibitory control. Their systematic review included pc-based music programs, instrumental training, multimodal music training, and theoretical instruction. Their research identified varying outcomes, which may be attributed to such factors as the study design and the length of the training period. Sala and Gobet (2020) undertook a systematic evaluation of the influence of music training on cognitive and academic performance in children using a multilevel meta-analysis. The studies that were incorporated into that analysis encompassed various forms of instrumental learning, the Kodály method, cognitive assessments unrelated to music, and measures of academic achievement. The results indicated that the overall effect sizes that were associated with music training on children’s cognitive and academic outcomes were minimal. Furthermore, these effects showed considerable diminishment and ceased to show statistical significance when the analysis was restricted to studies that employed active control groups or randomized nonactive control groups. Further, Bigand and Tillmann (2022) explored the impact of music training on cognitive processes, with a particular emphasis on both distant and near transfer effects. They indicated notable distant transfer effects associated with music training that are in contrast to those presented by Sala and Gobet (2020) findings. Taken as a whole, these studies cover a wide range of ages. However, the preschool years, from ages 3 to 6 years, are a pivotal phase for the development of children’s EFs. Interventions that are implemented during this period can substantially impact children’s cognitive abilities and their overall developmental trajectory (Best and Miller, 2010; Zelazo et al., 2003). Degé and Frischen (2022) suggested that music training may enhance EFs by providing children with heightened cognitive stimulation. Furthermore, Wiebe et al. (2008) proposed that inhibitory control is the most significant EFs to develop during preschool. Over the past few years, researchers have explored different aspects of EFs that are influenced by music training (Shen et al., 2019; Bowmer et al., 2018; Zou, 2021). However, the evidence is somewhat conflicting. A previous study showed that short-term music training enhances inhibitory control in preschool children (Moreno et al., 2011). However, other studies have indicated that short-term music training does not improve inhibitory control in preschool children (Bugos and DeMarie, 2017; Janus et al., 2016). This discrepancy may be attributed to various factors, such as the training program and testing task used.

While a considerable body of research has examined the influence that music training has on EFs, meta-analyses that specifically address the effects of music training on EFs in preschoolers aged 3–6 years are notably absent. Further, previous meta-analyses have not synthesized the three dimensions of EFs. Thus, this study focuses on preschool-aged children aged 3–6 years to explore the effects of music training on inhibitory control, working memory, and cognitive flexibility in a thorough meta-analysis. The music training activities investigated in this study include singing, playing instruments, composing music, and practicing pitch and rhythm, which are all typically incorporated into preschool curricula and are not generally considered to constitute specialized training. Previous research has found that these activities can positively influence children’s cognitive development. This study conducts subgroup analyses to assess the frequency per week, duration of each lesson, and total number of weeks of interventions to determine how these training variables affect EFs. The objective is to determine the relationship between the intensity of the music training and the enhancement of EFs. Using this methodological approach, this study identifies the specific effects of various training parameters, ascertains the most effective training methods, and provides tailored recommendations concerning educational practices. In addition, this study fills the gaps in the literature and provides empirical support for preschool education.

## Methods

This systematic review and meta-analysis was conducted according to the guidelines established by the Preferred Reporting Items for Systematic Reviews and Meta-Analyses (PRISMA) (Page et al., 2021). The study protocol was registered with the International Prospective Register of Systematic Reviews (No. CRD42024513482).

### Literature search

The publication databases Web of Science, PubMed, Scopus, and China National Knowledge Infrastructure were searched from their inception until September 9, 2023. The search terms used in English were “musical activity” OR “music program” OR “music course” OR “music curriculum” OR “music subject” OR “music training” AND “preschool children” OR “early children” OR “kindergarten” OR “preprimary children” OR “nursery school” OR “infants’ school” OR “young children” AND “executive function” OR “working memory” OR “inhibitory control” OR “cognitive flexibility.” The Chinese search terms were “音乐” AND “学前儿童” OR “幼儿” OR “儿童” AND “认知” OR “记忆” OR “注意” OR “抑制控制” OR “执行功能.” The works cited in previous reviews and other selected studies were screened to locate additional related studies.

### Eligibility criteria

Following the PICOS model (Saaiq and Ashraf, 2017), studies were considered eligible for inclusion in this review if they satisfied the following criteria: (1) study population of healthy preschool children aged 3–6 years; (2) intervention: music training in a group, including singing, playing, composing, practicing pitch, and practicing rhythm; (3) passive control group or other intervention group unrelated to music training, such as exercise group; (4) at least one EFs outcome variable, such as inhibitory control, working memory, and cognitive flexibility; (5) and in the form of a randomized or nonrandomized comparative training study. Studies were excluded if any of the following criteria were met: (1) participants were children under the age of 3 years or over the age of 6 years; (2) in the form of a literature review or meta-analysis; (3) not written in Chinese or English; (4) and concerning specialized instrumental or vocal music training.

### Study selection and data extraction

The first author imported the studies into Endnote X9 software (Clarivate Analytics, Philadelphia, PA, USA) and deleted duplicates. The first and second authors independently conducted preliminary screening, reviewing the titles and the abstracts of the studies before retrieving and assessing the full texts for eligibility. Disagreements concerning eligibility were addressed by consultation with the third author. The data extraction from the included studies was performed using an Excel spreadsheet, obtaining the following information (1) authors’ names, years of publication, regions of investigation; (2) samples’ sizes, ages, and genders; (3) length of intervention period, frequency of interventions per week, duration of one intervention, type of intervention; (5) and assessment task. Means and standard deviations of the pre- and posttests were extracted from the text. Where data were solely presented in figures, WebPlotDigitizer (version 4.5) software was employed to extract the data.

### Study quality assessment

Cochrane Collaboration was used to assess the quality of each study (Higgins and Green, 2008). The evaluations of all the studies analyzed were conducted independently by the first and second authors. Discrepancies in evaluation were resolved in discussion or by seeking input from the third author.

### Statistical analysis

A random-effects meta-analysis for each outcome was performed using Stata 16.0 software (Stata, TX, USA). According to the recommendation for effect size calculation in pre- and posttest intervention studies in meta-analyses (Morris, 2008), for each group, the mean difference was calculated as M_post_ − M_pre_, and the standard deviation used was SD_pre_. Because the outcome measures varied in different publications, standardized mean differences (SMDs) calculated using Cohen’s *d* and 95% confidence intervals (CI) were calculated. The SMD values were interpreted as follows: 0.2–0.49 were considered small, 0.5–0.79 were moderate, and >0.8 were large (Cohen, 1988). Heterogeneity was assessed using *I*^2^ statistics, where 25, 50, and 75% represented low, medium, and high ratios for inter-study heterogeneity, respectively (Higgins et al., 2003). For studies that employed different task measures for a given outcome (Shen et al., 2019; Bowmer et al., 2018; Bugos and DeMarie, 2017; Frischen et al., 2019), the most commonly used task measure was used in the calculation. After the overall effect size was calculated for each outcome, subgroup analyses were conducted for the intervention period, frequency per week, and duration per session. Publication bias was assessed using funnel plots, and Egger’s test was calculated with 95% CIs.

## Results

### Literature search

**Figure 1:**
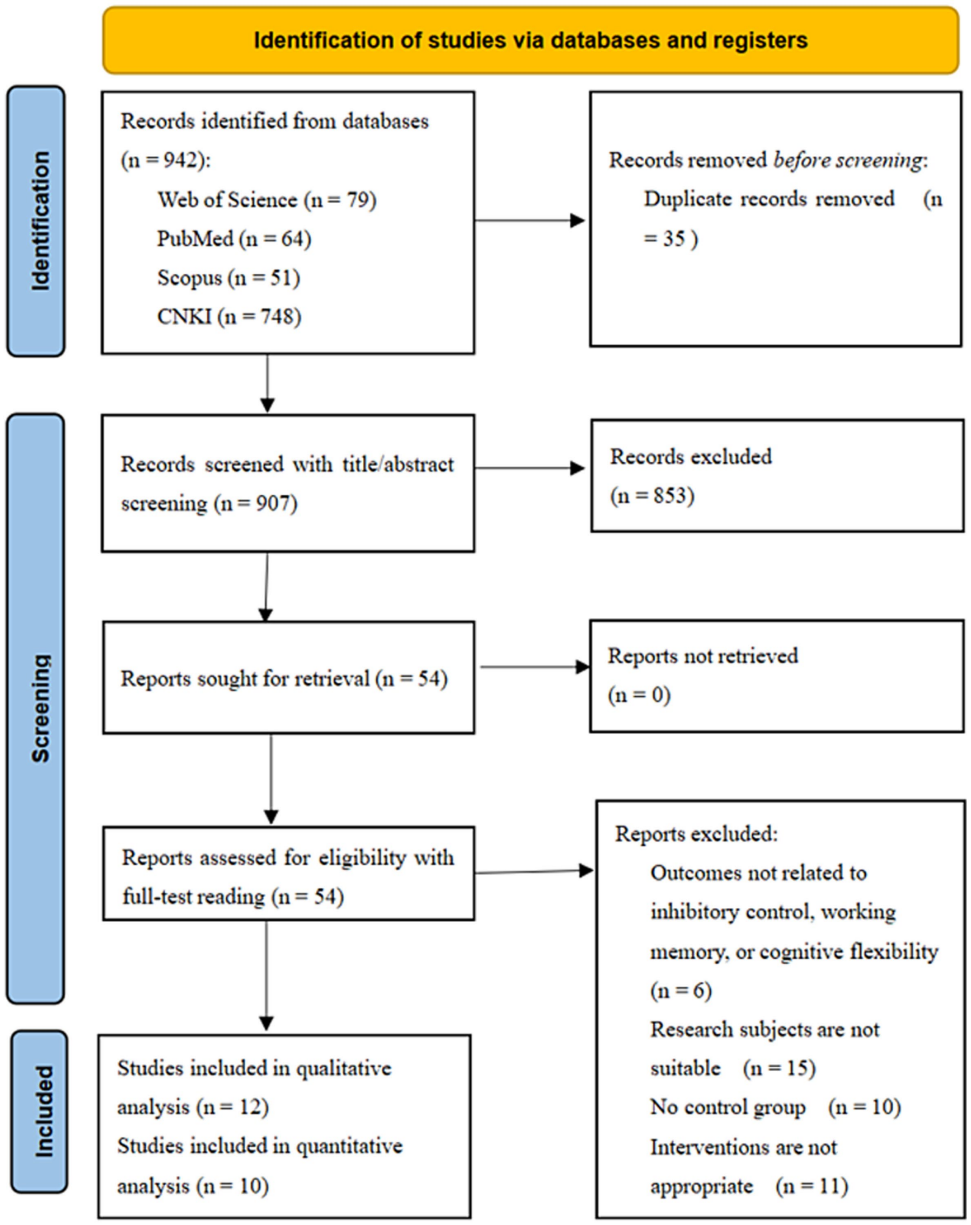
Flowchart of the literature screening process. Adapted from the PRISMA statement (Page et al., 2021).

Figure 1 presents the flow of the literature search and the screening process. In all, 942 articles in English and Chinese were retrieved. After 35 duplicate articles were removed and 907 articles were screened by the titles and abstracts, 54 articles were assessed for eligibility. The remaining 54 articles were read in full text. After this assessment, 12 studies were included in the systematic review, with Bolduc et al. (2021) and Moreno et al. (2011) included in the qualitative analysis due to insufficient data. The other 10 studies were included only in the quantitative analysis. Specifically, Bolduc et al. (2021) did not report means and standard deviations for the pre- and post-tests, and Moreno et al. (2011) did not provide the standard deviation for their raw data.

### Study characteristics

Table 1 presents the characteristics of all 12 studies that were included in this review. The studies originated in the United States (4), Germany (2), China (3), the United Kingdom (1), and Canada (2), with a total sample size of 807, including 449 participants in experimental groups and 358 in control groups. Each study included both male and female participants. The majority of studies showed a training frequency of 1–3 times per week. However, Shen et al. (2019) and Janus et al. (2016) had training frequencies of five times per week and every day of the week, respectively. In most studies, the intervention period lasted for 4–12 weeks. Notable, the study of Bolduc et al. (2021) was conducted over 19 weeks. Similarly, Frischen et al. (2019) conducted their study over 20 weeks, while Rauscher and Zupan (2000) conducted their interventions over 4–8 months. The studies by Frischen et al. (2019) (rhythmic group and pitch group), Zou (2021) (rhythmic group and pitch group), and Bolduc et al. (2021) (music group and motor group) each involved two experimental groups. Six studies assessed inhibitory control, working memory, and cognitive flexibility, while four focused on inhibitory control alone.

**Table 1:** Basic characteristics of included studies.

Study	Region	Age (years)	Subjects (E/C)	Duration and frequency	Period	Experimental group	Control group	Assessment task	Qualfications of educator
Bolduc et al. (2021) (qualitative assessment)	Canada	5–6	50, 52/58	40 min/session, 1 time/week	19 weeks	The music (E1) and motor (E2) conditions comprised six themes: (1) fall, (2) forest, (3) holiday festival, (4) trip around the world, (5) imaginary world, and (6) circus. Music activities (E1): inspired by the American Music Playback program and Orff’s active music participation approach. Motor activities (E2): draw on the basic concepts and instructional applications of motor skills as defined by Deitz et al. (2007).	Creative activities	NEPSY-II (Inhibition control)	Two music education doctoral students conducted musical activities under the guidance of an expert in the field. A team of eight undergraduates majoring in kinesiology collaborated on the development of exercise activities under the guidance of a master’s student and a professor of kinesiology.
Bowmer et al. (2018)	United Kingdom	3–4	E1: 13/24 E2: 13/11	40 min/session, 1 time/week	8 weeks/16 weeks	The research was carried out in conjunction with the London-based charity Creative Futures, which specializes in providing high-quality music and arts programs, often with a pre-school focus. Pitch imitation, melody recognition, musical phrasing, and musical anticipation.	C1: Regular activities. C2: Art class: courses are based on different techniques and are themed on the work of specific artists.	Peg Tapping (Inhibition control) Baby Stroop (Inhibition control) Spin the Pots (Working memory) DCCS (Cognitive flexibility) Trucks (Cognitive flexibility)	All music intervention sessions in both phases are led by the same Creative Futures Practitioner. The art program is designed and taught by Creative Futures’ early professional art practitioners.
Bugos and DeMarie (2017)	United States of America	4–5	17/17	45 min/session, 2 times/week	6 weeks	Through music education pedagogies such as Orff-Schulwerk and Kodály, creative activities, focusing on large-muscle motor coordination through the use of electronic and acoustic instruments (African drums, xylophones, and iPads), vocal development exercises, and improvisation activities.	LEGO training: training in building different shapes/numbers, forming patterns, sorting and creative exploration.	Day/Night Stroop Task (inhibition control)	All music courses are taught by university professors with 17 years of teaching experience and a Kindermusik-accredited doctorate in music education, and by doctoral-level music education students with more than 20 years of experience teaching young children. A trained research assistant with expertise in early childhood manages the LEGO program.
Cai (2023)	China	5	32/32	30 min/session, 2 times/week	8 weeks	This study generally followed the types of musical games used in Kosokabe’s study and Bowmer’s components on the level of musical games, referred to the Executive Function Training Methods of “70 Play Activities for Children I Promote the Development of Thinking and Executive Functions,” and used the Guidelines for Kindergarten Education (Trial), the Guidelines for Learning and Development of Children 3–6 Years of Age, and Xu Zhuoya’s “Musical Education for Preschool Children” as the basis for adapting a musical play program suitable for the development of executive function in middle-grade children. Include: rhythmic games, involving pitch, rhythm, melody, acting games, singing games, percussion games, and listening games.	Regular activities	Go/No-Go (Inhibition control) Mr. Ant (Working memory) Card Sorting (Cognitive flexibility)	Master of Music
Degé et al. (2022)	Germany	5–6	11/14	20 min/session, 3 times/week	14 weeks	Singing, drumming, as well as dancing to a variety of songs and playing different percussion instruments.	Exercise in balance, strength, endurance, coordination, fine motor skills, body perception, and relaxation.	NEPSY-II (Inhibition control)	Both types of training are based on written agreements provided to trainers in the form of manuals.
Frischen et al. (2019)	Germany	5–6	33/33/30	20 min/session, 3 times/week	20 weeks	The training sessions were implemented in the manner described in the study by Patscheke et al. (2018) and based on a well-established early music education program designed by Nykrin et al. (2007) E1: Rhythmic Training, incorporating vocal gestures (clapping and stomping) and Orff instruments. E2: Pitch training, incorporating tone recognition, intonation, vocalization, and joint singing.	Exercise: balance, strength, endurance and relaxation.	NEPSY-II (Inhibition control) Matrix Span Test (Working memory) Corsi Block Test (Working memory) DCCS (Cognitive flexibility)	The training program is based on the manual. Each week, research assistants and research supervisors meet to prepare and practice for the following week’s training session.
Ilari et al. (2021)	United States of America	4–6	51/52	40 min/session, 2 times/week	20 days	The program was designed for this study based on a curriculum developed earlier by the Creative Futures team: Seven main parts to each lesson: (1) “hello” song, (2) action song, (3) playing instrument, (4) movement activities, (5) songs and instrumental music using props, (6) relaxation activities, (7) “goodbye” song.	Reading, coloring, arts and crafts, and group projects (e.g., jigsaw puzzles).	Spin the Pots (Working memory /Inhibition control) DCCS (Cognitive flexibility)	Music courses are taught by music teachers (i.e., college students majoring in music).
Janus et al. (2016)	United States	4–6	29/28	2 h/session per day	5 weeks	A combination of rhythm, pitch, melody, voice, and basic musical concepts.	French language training, including vocabulary, communication scenarios.	Word Span (Inhibition control) Corsi Block Test (Working memory)	Two teachers specializing in French or music education.
Moreno et al. (2011) (qualitative assessment)	Canada	4–6	24/24	45 min/session, 2 times/day, 5 days/week	4 weeks	Two computerized training programs (both created by Sylvain Moreno) were administered. This training relies heavily on listening activities rather than instrumental training: A combination of rhythm, pitch, melody, voice, and fundamental musical concepts.	Visual art curriculum: developing visuospatial skills, such as shape, color, line, dimension, and perspective concepts.	Go/No-Go (Inhibition control)	One teacher leads the group in the activity.
Rauscher and Zupan (2000)	United States of America	5–6	34/28	20 min/session, 2 times/week	4 months/8 months	Ear training, notation, rhythm, improvisation, intervals, and dynamic exercises.	Regular activities	Pictorial Memory (Working memory)	Music specialist
Shen et al. (2019)	China	4	30/31	45 min/session, 5 times/week	12 weeks	Using an integrated approach to musical training, the songs selected for the experiment were from John Thomson’s Modern Piano Course (WILLIS, Shanghai Music Press), and the researchers created their own repertoire based on the teaching content: A combination of movement, sensory, and cognitive tasks, encompassing training in rhythm, pitch, melody, sound, and fundamental music concepts.	Free play	Day/Night Stroop Task (Inhibition control) Backward-Digit Span Task (Working memory) Dot Matrix Test (Working memory) DCCS (Cognitive flexibility)	Master’s Degree in Musicology.
Zou (2021)	China	3–6	20/20/20	20 min/session, 3 times/week	12 weeks	Following the training program used in the study by Frischen (2019) and adapted and improved by researchers such as Patscheke (2018) on the basis of the early music education program designed by Nykrin (2007), as well as referring to the rhythmic training based on Orff’s ideas on music education and the aural training components based on Kodály’s ideas on music education: E1: Pitch training, enabling children to recognize the pitch of sounds made by different objects, instruments and people. E2: Rhythmic training, enabling children to perceive, imitate and create rhythms using Orff instruments and vocal gestures.	Regular activities	Go/No-Go (Inhibition control) Mr. Ant (Working memory) Card Sorting (Cognitive flexibility)	Master of Music

DCCS, dimensional change card sort.

NEPSY-II, NEuroPSYchological Assessment Second Edition.

C1, control group 1.

C2, control group 2.

E1, experimental group 1.

E2, experimental group 2.

### Risk of bias assessment

**Figure 2:**
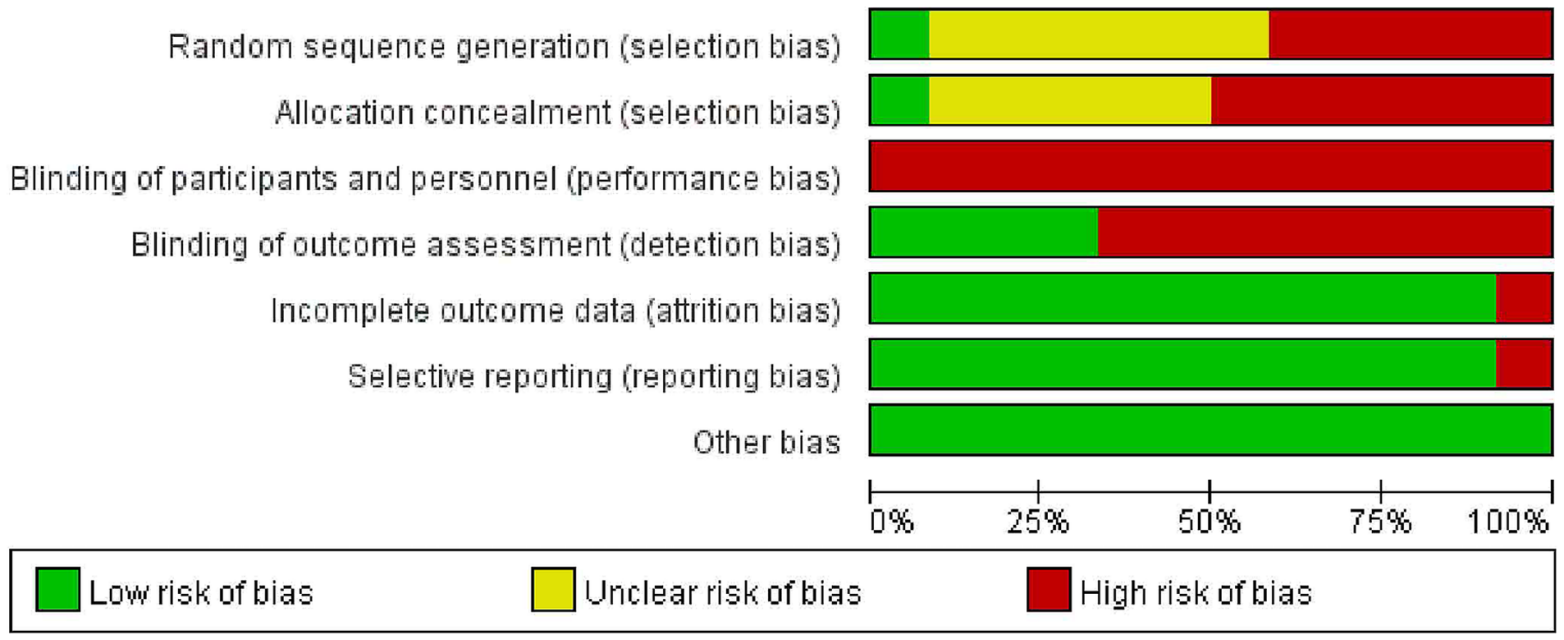
Risk of bias graph.

The risk of bias assessment is presented in Figure 2. Only one study described how a randomized sequence was generated, three studies referred to “pseudo-randomization,” and six studies referred to “randomization” without providing details on the particular method used. One study employed centrally randomized allocation, while the remaining studies provided no description of the concealment of allocation. Additionally, due to the nature of the intervention, it was not feasible to blind subjects, producing a high risk of bias in all studies. Blinding was provided for outcome measures in four studies, while 11 studies reported complete outcome data, and one had incomplete outcome data. Furthermore, all studies exhibited low selectivity bias. No other biases were noted.

In training intervention studies, subjects are made explicitly aware of whether they are receiving the experimental intervention. This can lead to performance bias in which subjects’ behavior is influenced by their knowledge of the intervention that they are receiving. However, in children aged 3–6 years, this bias may have a smaller impact due to their limited understanding of the purpose of the intervention. In addition, the lack of detailed descriptions for the generation of random sequences generation and allocation concealment poses a significant risk for selection bias. These biases can affect the validity and reliability of the outcomes of the study. It is essential to address these issues in future research through more rigorous methodological practice to enhance the robustness of findings in this field.

### Meta-analysis

#### Inhibitory control

**Figure 3:**
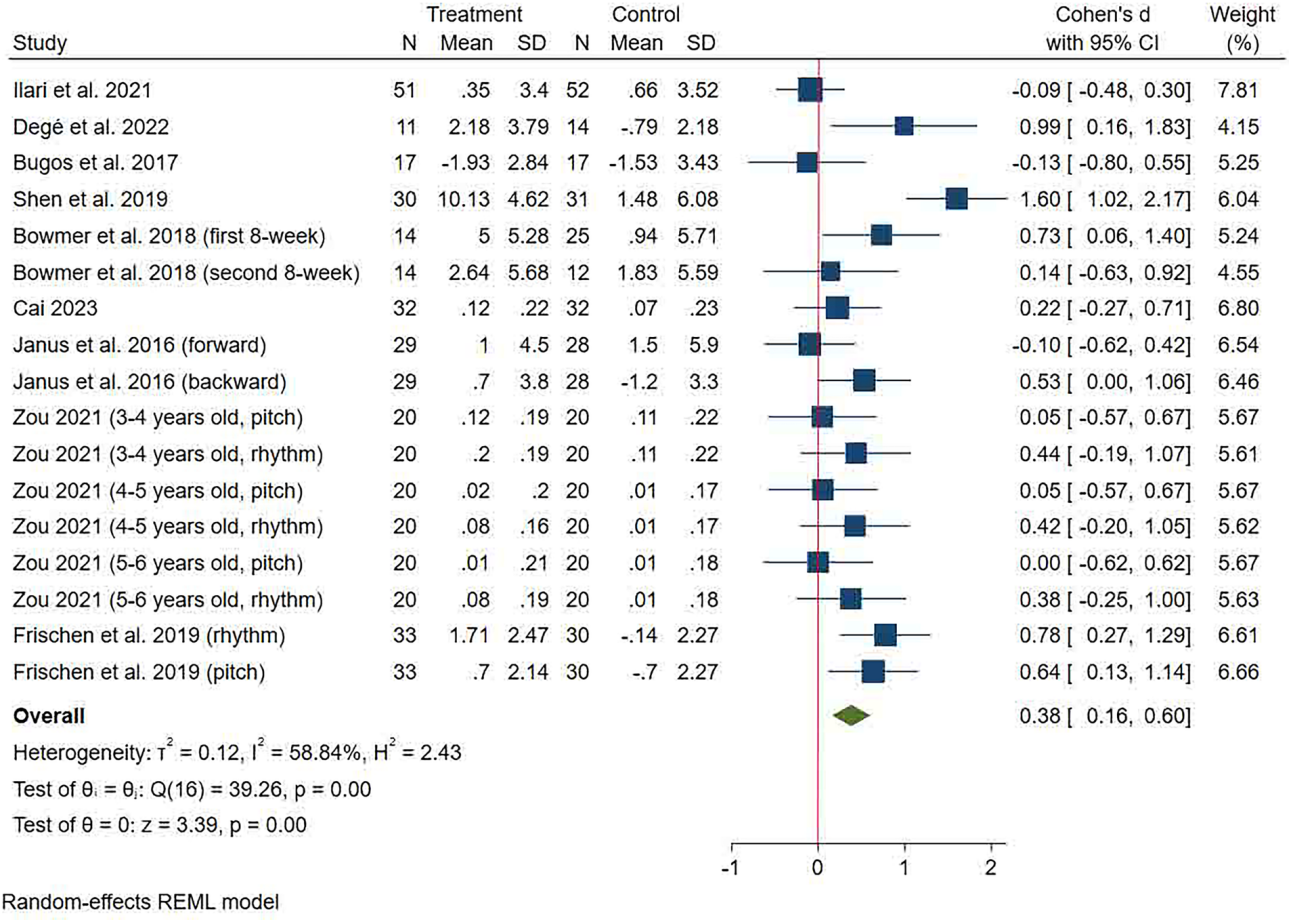
Forest plot of meta-analysis of inhibitory control.

Nine studies assessed inhibitory control for a total of 17 effect sizes (Figure 3). The pooled effect size showed that music training significantly improved children’s inhibitory control relative to the control group (SMD = 0.38 (0.16, 0.60), *p* < 0.001). Significant heterogeneity was observed between studies (*I*^2^ = 58.84%, *p* < 0.001). Egger’s test revealed no significant publication bias (*p* = 0.353, 95% CI: −2.46, 6.49, the funnel plot is in Supplementary material). The results of subgroup analysis showed that music training significantly improved children’s inhibitory control for a duration of ≥12 weeks, ≥3 times per week, and 20–30 min per session relative to the control group (Table 2).

**Table 2:** Subgroup analysis of executive functions.

Outcome	Moderator	Subgroup	*N*	Heterogeneity test results			Meta-analysis results		
				*Q*	*p*	*I*^2^/%	SMD	95%CI	*p*
Working memory	Weeks	<12	5	8.71	0.069	54.1	0.21	[−0.12, 0.55]	0.218
		≥12	12	20.87	0.035	47.3	0.42	[0.18, 0.65]	<0.001
	Frequency (week/times)	<3	6	19.29	0.002	74.1	0.49	[0.06, 0.93]	0.027
		≥3	11	11.98	0.286	16.6	0.28	[0.1, 0.47]	0.003
	Duration (min)	20–30	11	17.98	0.055	44.4	0.39	[0.16, 0.54]	0.001
		31–40	3	5.69	0.319	12.5	0.01	[−0.33, 0.35]	0.962
		>40	3	2.28	0.058	64.8	0.51	[−0.01, 1.02]	0.053
Cognitive flexibility	Weeks	<12	3	8.64	0.013	76.8	0.2	[−0.41, 0.81]	0.522
		≥12	10	8.56	0.479	0	0.22	[0.04, 0.41]	0.019
	Frequency (week/times)	<3	4	9.36	0.025	68	0.3	[−0.19, 0.8]	0.231
		≥3	9	7.09	0.527	0	0.19	[0.0, 0.39]	0.047
	Duration (min)	20–30	8	2.1	0.954	0	0.11	[−0.1, 0.31]	0.303
		31–40	4	9.36	0.025	68	0.3	[−0.19, 0.8]	0.231
		>40	1	Not applicable			0.74	[0.22, 1.26]	0.005
Inhibitory control	Weeks	<12	6	7.91	0.161	36.8	0.16	[−0.11, 0.44]	0.235
		≥12	11	24.08	0.007	58.5	0.51	[0.22, 0.8]	0.001
	Frequency (week/times)	<3	5	4.95	0.293	19.1	0.13	[−0.15, 0.41]	0.367
		≥3	12	28.31	0.003	61.1	0.48	[0.2, 0.75]	0.001
	Duration (min)	20–30	9	8.86	0.354	9.7	0.42	[0.2, 0.63]	0.001
		31–40	4	4.39	0.222	31.7	0.19	[−0.48, 0.3]	0.272
		>40	4	22.21	<0.001	86.5	0.48	[−0.3, 1.26]	0.225

N, number of effect sizes.

#### Working memory

**Figure 4:**
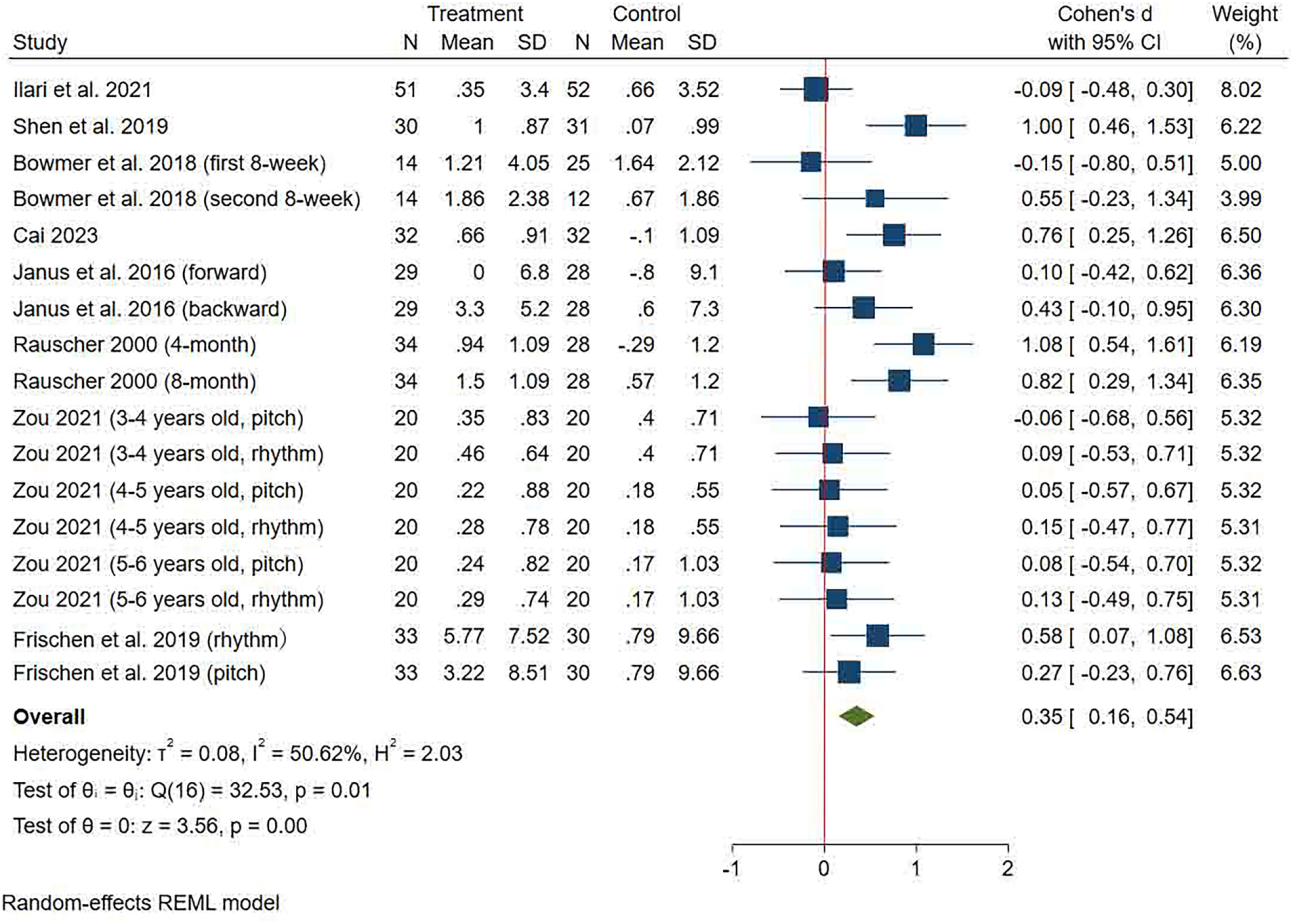
Forest plot of meta-analysis of working memory.

Working memory was assessed in eight studies, and a total of 17 effect sizes were included in the analysis (Figure 4). The pooled effect size demonstrated that music training significantly improved children’s working memory relative to the control group (SMD = 0.35 (0.16, 0.54), *p* < 0.001). A moderate heterogeneity was seen between the studies (*I*^2^ = 50.62%, *p* < 0.001). Egger’s test indicated that there was no significant publication bias (*p* = 0.903, 95% CI: −5.2, 4.63, the funnel plot is in Supplementary material). Subgroup analysis showed that the music training group significantly improved children’s working memory over ≥12 weeks and 20–30 min per session relative to the control group, irrespective of training frequency (Table 2).

#### Cognitive flexibility

**Figure 5:**
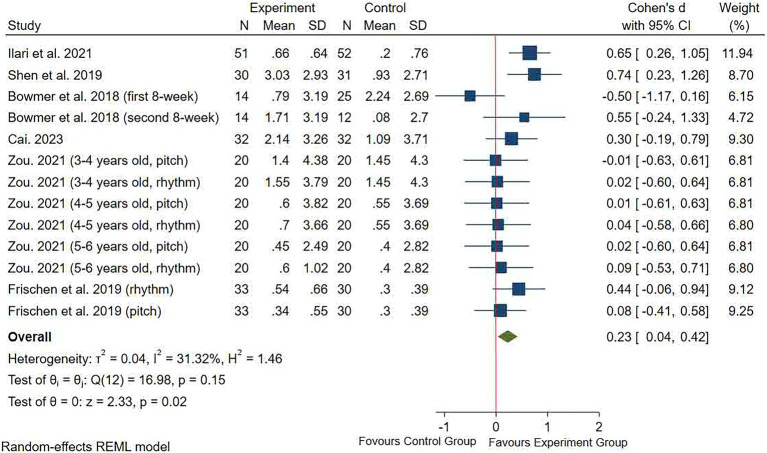
Forest plot of meta-analysis of cognitive flexibility.

Cognitive flexibility was examined in six studies with 13 effect sizes that were included in the results (Figure 5). The pooled effect size showed that music training significantly improved children’s cognitive flexibility relative to the control group (SMD = 0.23 (0.04, 0.42), *p* = 0.02). A low level of heterogeneity was seen between the studies (*I*^2^ = 32.75%, *p* = 0.13). Egger’s test showed no significant publication bias (*p* = 0.057, 95% CI: −7.38, 0.13, the funnel plot was shown in Supplementary material). The results of the subgroup analysis indicated that the music training group significantly improved children’s cognitive flexibility over a duration of ≥12 weeks, ≥3 times per week, and ≥40 min per session, relative with the control group (Table 2).

## Discussion

This systematic review and meta-analysis is the first to examine the effects of music training on inhibitory control, working memory, and cognitive flexibility in preschool children aged 3–6 years relative to controls. We found that music training had a significant and positive effect on children’s EFs, a finding that has important implications for the field of early education. Further, through subgroup analyses, this study elucidated the varying impacts that distinct music training programs have on children’s EFs. The results indicate that variations in training duration and frequency produce significantly different outcomes for the enhancement of specific components of EFs. This evidence provides an empirical foundation for the development of tailored and effective music education programs. These findings not only address a gap in the literature on the influence of music training with respect to the EFs of preschool children but also offer critical insights for the formulation of future educational policy and guiding future interventions.

### Inhibitory control

A meta-analysis demonstrated a significantly and positive effect of music training on inhibitory control in preschool children relative to controls (SMD = 0.38). Subgroup analyses revealed that longer music-intervention periods (≥12 weeks) are more beneficial for improving inhibitory control. It is worth noting that one study showed an improvement in the go/no-go task following 4-week music training, accompanied by increased P2 wave amplitude. The authors suggest that improvement in inhibitory control tasks may be attributed to the simplicity of the go/no-go task (Moreno et al., 2011). By contrast, other studies have found that short-term music training does not significantly improve inhibitory control in preschool children (Ilari et al., 2021; Bugos and DeMarie, 2017; Janus et al., 2016), which may be related to the use of more difficult tasks [e.g., spin the pots (Ilari et al., 2021) and word span (Janus et al., 2016)]. Taking into consideration that preschool children are very active and may struggle to inhibit active behavior, it may be difficult to effectively measure children’s inhibitory control with the use of more complex tasks.

Research has shown that children from lower socioeconomic backgrounds frequently experience cognitive difficulties due to limited educational resources and persistent stress (Noble et al., 2005; Hackman et al., 2010). Degé et al. (2022) found that music training significantly improved inhibitory control in children from low-income families. Similarly, Bugos and DeMarie (2017) reported enhancements in verbal and visuomotor inhibitory control tasks among children from high-income families who participated in music training. These findings highlighted the potential of music training as an effective intervention for improving inhibitory control, regardless of socioeconomic status. Thus, music training emerges as a promising intervention for enhancing inhibitory control in preschoolers from various socioeconomic backgrounds.

The development of inhibitory control can also be affected by children’s age. For instance, Cai (2023) reported no positive effects for a go/no-go task among preschool children aged 4–6 years after music training. Zou (2021) also found that the pitch group did not demonstrate statistically significant improvements in inhibitory control compared to the control group across all age groups. However, children aged 3–4 years showed a significant within-group improvement, while the 4–5 and 5–6 year age groups did not. This phenomenon may be linked to the pivotal phase of cognitive development that is experienced by children aged 3–4 years. In this stage, children present significant progress in emotional self-regulation and social integration abilities and show an increased openness to EFs training. Furthermore, Frischen et al. (2019) observed that rhythm and pitch training significantly improved inhibitory control in children aged 5–6 years compared to the control group. Degé et al. (2022) reported significant enhancements in inhibitory control in children from 5 to 6 years old group after they engaged in musical training, which contrasts with the lack of improvement that was seen in the control group. This current study provides further evidence with respect to the efficacy of music training in enhancing the inhibitory control in specific developmental stages. The findings show the complex interplay between the maturation of children’s physiological and psychological functions as well as the influence of external training and educational interventions on the development of inhibitory control. It is crucial to acknowledge age-specific variations such as these when designing customized educational and training programs. These programs can assist educators and researchers in formulating more effective strategies to improve EFs in children across age groups while also providing a scientific basis for future educational methods. The current results with respect to children aged 5–6 years have exhibited inconsistencies across various studies. Future research should examine the effects of music training in this age group more closely to comprehensively evaluate and enhance educational interventions.

### Working memory

Meta-analysis showed the effectiveness that music training has for improving working memory in preschool children relative to a control group (SMD = 0.35). Subgroup analyses indicate that durations of ≥12 weeks and 20–30 min per session were advantageous for producing improvements in working memory. In particular, one study reported no improvements in working memory following a 5-week music training (Janus et al., 2016). The authors found that the lack of improvement in working memory could be attributable to the challenges of the task and the abbreviated intervention duration.

The impact of music training on working memory varies according to the type of training and the assessment tasks used. Frischen et al. (2019) compared the effects of 20 weeks of rhythm, pitch, and motor training on working memory, assessed using two tasks: the Matrix Span and the Corsi Block. The current study included the Corsi Block outcomes in the meta-analysis. The results indicated that while the rhythm training group showed significant improvements compared to the control group, the pitch training group did not demonstrate significant differences. Notably, the rhythm training group exhibited a decline in the Matrix Span test compared to the baseline, whereas the pitch training group improved both assessment tasks. These findings suggest that different types of music training may have distinct impacts on working memory. However, Zou (2021) conducted a 12-week training intervention, using the Mr. Ant task for evaluation, and found no improvement for either a pitch training group or a rhythm training group compared to the control group. From the above, the inconsistency in the outcomes of rhythm training may be related to the assessment tasks employed. Additionally, this may be attributable to the complexity of rhythm perception, which is predominantly characterized by the distinction of durations. The distinction of durations are difficult to auditory sense; they can only be recognized after processing through abstract cognition (Zou, 2021). This phenomenon could be associated with the malleability of preschool children’s brains and the maturation of their working memory. In particular developmental stages, preschool children may not exhibit optimal responsiveness with respect to certain cognitive stimuli, which potentially limits the effectiveness of rhythm training. Furthermore, the structure and complexity of the rhythm training regimen may not be sufficiently challenging to enable engagement and improve the working memory capacity of preschool children. Although, in specific instances, rhythm training can improve working memory, its efficacy may not be consistent across all preschool children. Future studies should explore the variables that impact the efficacy of music training, including individual variance, the precise content and methodology of the training, and strategies for tailoring programs that can align them with children’s diverse developmental requirements.

### Cognitive flexibility

Meta-analysis showed the efficacy that music training had in improving preschool children’ cognitive flexibility relative to a control group (SMD = 0.23). Subgroup analyses indicated that longer music training duration (i.e., ≥12 weeks, ≥3 times per week, and ≥40 min per session) were optimal for improving cognitive flexibility. Bowmer et al. (2018) conducted a two-phase experiment. Following the initial 8 weeks, the magnitude of improvement in cognitive flexibility was greater in the control group compared to the music training group. After the second phase, although no significant differences in cognitive flexibility were observed between groups, the music group significantly improved from their mid-term performance and demonstrated notable enhancements relative to the control group, implying the potential contribution that long-term music training had to improvements in cognitive flexibility. Bowmer et al. (2018) noted that the lack of improvement in cognitive flexibility in the first 8 weeks of intervention may be attributed to the difficulty of the task, which was not immediately able to capture the attention of preschool children.

Cognitive flexibility tends to mature at a later stage than inhibitory control and working memory (Diamond, 2013). It has been posited that the progress of cognitive flexibility among preschool children is relatively constrained, with its primary developmental phase not occurring until later in the school year. For instance, utilizing the Dimensional Change Card Sort Task, Frye et al. (1995) examined cognitive flexibility. Their findings showed that 3-year-old children encountered difficulties in the completion of their tasks; by contrast, a significant improvement was observed at 4 years old relative to 3 years old. They also found that children did not exhibit proficient task performance until they reached 5 years old. Further, Davidson et al. (2006) demonstrated that, even when cognitive flexibility tasks involved simple demands, children required considerable time to reach levels that were comparable to those of adults.

The improvement in cognitive flexibility attributed to music training may be linked to the particular content of the training. A variety of activities incorporated in music course require children to swiftly shift and adjust among different musical elements, and this process of transitioning contributes to the enhancement of their cognitive flexibility. For example, children may need to make adjustments to the tempo of a song or changes to the lyrics when singing a song. Activities such as making different gestures based on musical elements, or taking turns singing or clapping. Such activities not only require children to respond quickly, but also promote their development of cognitive flexibility (Ilari et al., 2021).

Some limitations to this study need to be addressed. Gender differences were not examined in this study due to the lack of detail in the studies included. Previous research showed significant gender variations in EFs among preschool children (Veraksa et al., 2022). This should be examined in future studies. Additionally, the lack of uniformity in musical instruction techniques across studies is a limitation. The variations in musical training methods, including differences in teaching approaches and the complexity of activities, may have influenced the outcomes and contributed to the observed inconsistencies. Another limitation was the high risk of bias that was identified in the included studies. Random sequence generation and allocation concealment were not adequately described in most studies, which introduces a significant risk of selection bias. These biases could affect study outcomes and should be addressed in future research through more rigorous methodological practices.

## Conclusions and recommendations

Music training has positive effects on inhibitory control, working memory, and cognitive flexibility. All three of these subcomponents of EFs are most effective for training durations of ≥12 weeks and ≥3 times per week. Sessions that last 20–30 min show the most significant effects on inhibitory control and working memory, while sessions that exceed 40 min had the greatest impact on cognitive flexibility. However, the results of sessions exceeding 40 min should be interpreted with caution, as only one study used this duration.

In addition to the volume of training, other factors that influence the effects of music training on EFs should also be noted, such as types of tasks and music training content. Among the studies included in this meta-analysis, specific interventions varied in terms of duration, frequency, and content. Some studies focused on rhythm and pitch discrimination tasks, while others involved instrumental performance or integrated music training sessions. It is important to note that the effectiveness of these interventions was influenced not only by the content, but also by the pedagogical methods used. Different teaching methods, including the teacher’s style and interaction with the child, may significantly impact the results. For example, a teacher’s level of engagement, teaching strategies, and responsiveness to individual children’s needs can affect how well children respond to interventions. In rhythmic training, for instance, a teacher’s ability to maintain children’s attention and guide their learning through active participation may lead to more significant improvements in EFs. Therefore, teaching methods and teacher-student interactions should be considered when evaluating the effectiveness of music training programs, as these factors may contribute to variability in outcomes. Future research should aim to standardize teaching methods or more explicitly consider the role of the teacher to better understand the effects of different music training types on executive functions. Methodologically, only a limited number of studies have investigated the mechanisms between music training and EFs, such as the use of event-related potential and functional magnetic resonance imaging. Further study involving brain imaging techniques should be conducted to deeper into the neural underpinnings of the effect of music training on EFs, as well as the alterations in the structure and function of the brain resulting from music training.

## Data Availability

The original contributions presented in the study are included in the article/Supplementary material, further inquiries can be directed to the corresponding author.
