# Supplementary figures and images for: Effects of music training on executive functions in preschool children aged 3–6 years: systematic review and meta-analysis
Source: Front Psychol. 2025 Jan 15;15:1522962. doi: 10.3389/fpsyg.2024.1522962 (PMC11775157; doi:10.3389/fpsyg.2024.1522962)

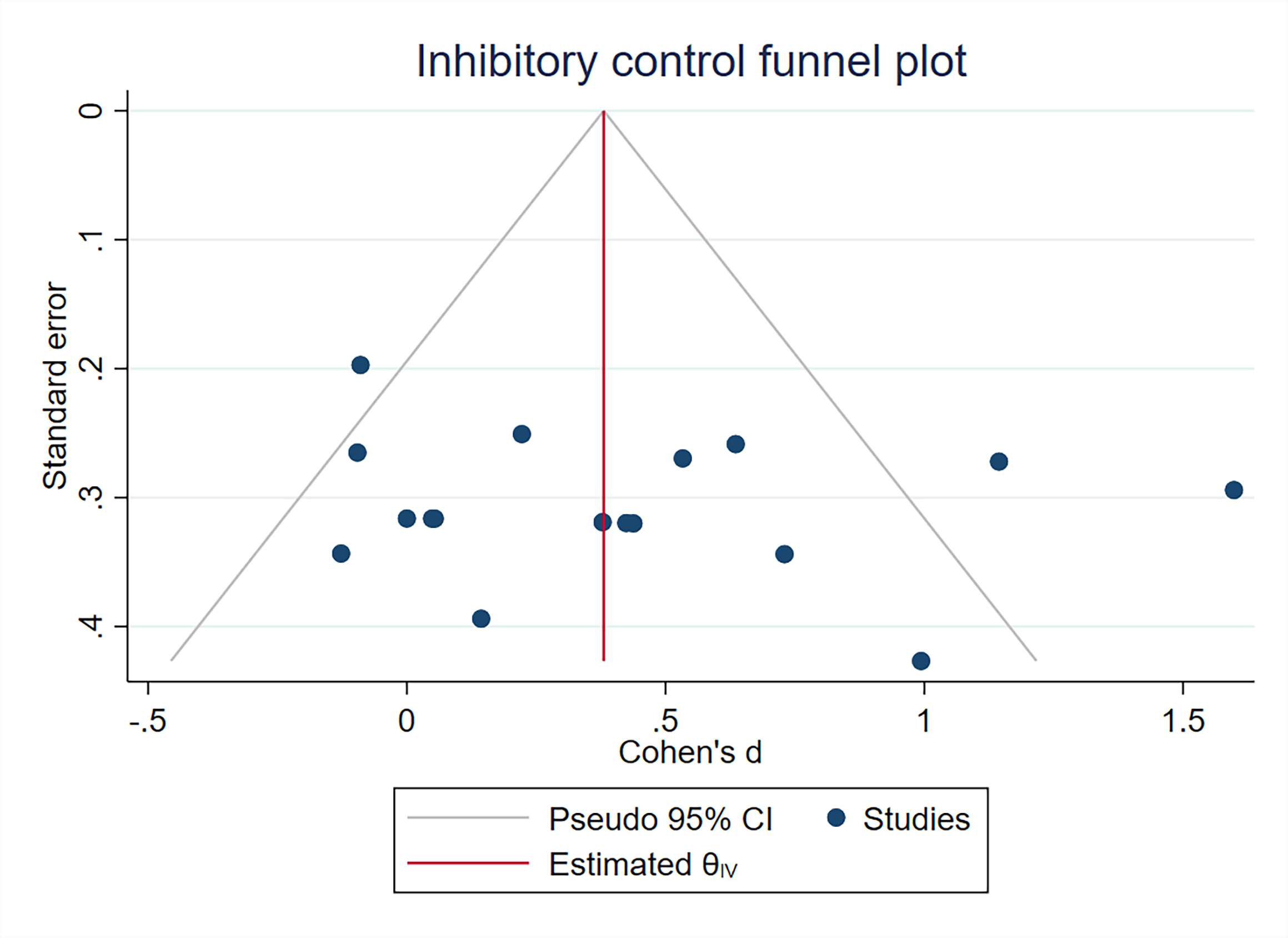

Supplement: Supplementary file 3 [file Image_1.TIF]

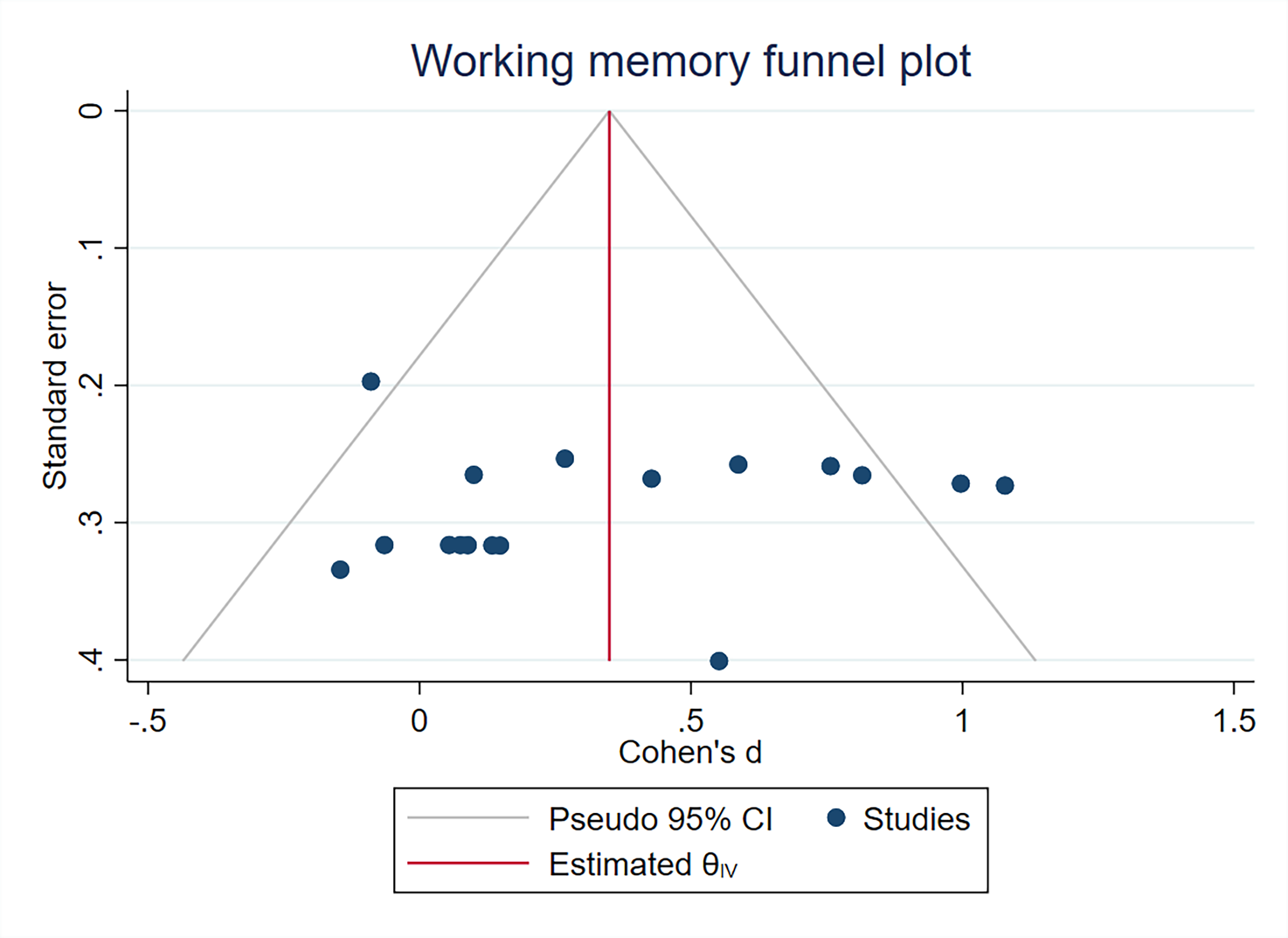

Supplement: Supplementary file 4 [file Image_2.TIF]

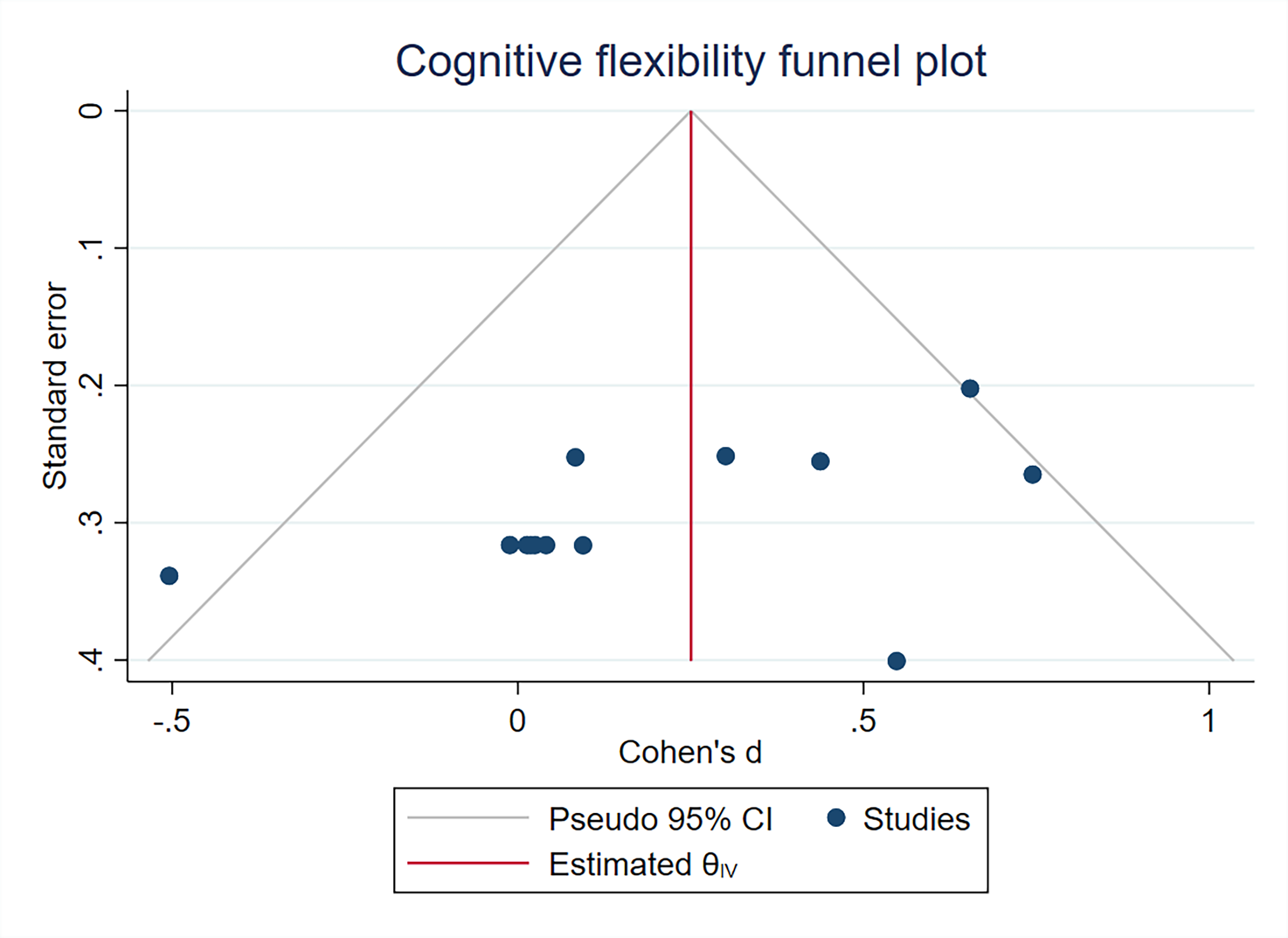

Supplement: Supplementary file 5 [file Image_3.TIF]
